# Books

**Published:** 1882-04

**Authors:** 


### Books.

---

A Treatise on Diseases of the Eye. By Henry D. Noyes, A.M., M.D. New York: William Wood & Co. 1881. Wood's Library of Standard Medical Authors.

Dr. Noyes, whom the reviewer recalls, always with pleasurable reflections since his days of pupilage in old Bellevue, more than a decade ago, and whom he naturally associates with the chair which the professor still graces, is the author of the work before us. He has attempted, so he says, to condense within the limits assigned him the substance of modern ophthalmic knowledge.

The reader is prepared to accept, with an unusual degree of confidence, the statements and opinions of the writer, after perusing the preface, in which he truthfully and forcibly affirms, "Nor can diseases of the eye be discussed without taking into account diseases of remote organs and of the general system. To them, therefore, brief reference is often made. Ophthalmology, as a field of special cultivation has, in late years, attained a wonderful development, but its relations to and dependence upon general pathology are more manifest now than ever. It has shed light of no feeble kind upon the pathology of other parts of the body, but it none the less seeks to be illuminated by knowledge procured from the study of general medicine."

If this view was entertained and these theories were practiced by all "specialists" it would undoubtedly very materially promote their usefulness and as surely shield them from many adverse, but generally just criticisms.

The book is divided into two parts. The first is devoted to disturbances of refraction and of muscular function. The second to inflammations and other structural changes. The work is well illustrated, and, so far as a casual examination enables us to determine, comes fully up to the standard marked out for it, and is clearly entitled to the place which it will occupy among the many excellent treatises composing this library. We do not hesitate to say that it will well repay our readers to invest the mere pittance asked—$1.25 per month—in this series of publications.

By way of parenthesis, we will venture to observe that the figure on page twenty-five is a very correct likeness of the accomplished writer.

